# Do pragmatic trials trade-off internal validity for external validity?

**DOI:** 10.1186/1745-6215-16-S2-O81

**Published:** 2015-11-16

**Authors:** Kirsty Loudon, Merrick Zwarenstein, Frank Sullivan, Peter Donnan, Shaun Treweek

**Affiliations:** 1 University of Aberdeen, Aberdeen, UK; 2 University of Dundee, Dundee, UK; 3 Western University, London, Canada; 4 University of Toronto, Toronto, Canada

## 

Trials can be described as being on a design spectrum between highly explanatory (roughly, ‘Can the intervention work?’) to highly pragmatic (‘Does the intervention work in routine care?’). A criticism levelled at trials that take a pragmatic approach is that they sacrifice internal validity for external validity, i.e. there is a trade-off to be made. Proponents of pragmatic trials argue that there is no trade-off. However, both critics and defenders of the pragmatic approach have made their arguments in the absence of empirical evidence one way or the other.

As part of our work developing the PRECIS-2 trial design tool, we looked for evidence of the validity trade-off in two ways. Firstly, a sample of 14 cardiovascular explanatory trials was matched to trials of the same intervention but which took a more pragmatic design approach. Secondly, 23 trials included in a Cochrane review of first-line treatment for hypertension were compared. The Cochrane Risk of Bias tool was used to assess internal validity; PRECIS-2 was used to assess design approach.

We found no clear difference in Cochrane Risk of Bias assessments between trials taking explanatory and pragmatic approaches. Work with a larger sample of trials is required before we can be completely confident in this result but this represents the first evidence that the suggested trade-off between internal and external validity in trials may be false.

This work is part of the Trial Forge initiative to improve trial efficiency.

